# Clinical impact of biologic therapy in the treatment of SLE

**DOI:** 10.1186/ar4223

**Published:** 2013-07-11

**Authors:** Daniel J Wallace

**Affiliations:** 1 Clinical Professor of Medicine, Cedars-Sinai Medical Center, David Geffen School of Medicine at UCLA, Los Angeles, CA, USA

## 

Biologic therapies have generated substantial research interest for treating autoimmune diseases, resulting in several US Food and Drug Administration approvals. However, clinical trial results in systemic lupus erythematosus (SLE) populations have failed to meet efficacy expectations, primarily owing to methodological flaws in design, especially outcome measures. To address these issues, the US Food and Drug Administration published a guidance document for clinical trial design in SLE that included specific efficacy endpoints for measuring disease severity. Belimumab was the first biologic approved for SLE based on positive responses measured with the SLE Responder Index, which required a decrease in inflammation, no new organ domains being involved, and improvement in physician global assessment. Trials of other biologics have used other versions of SLE Responder Index responder indices. In addition to belimumab, a score of other biologic agents are being investigated, primarily those targeting T cells, B cells, complement, and tolerogen-targeting agents. Their development stages and clinical trial results vary. Belimumab has generated the greatest amount of clinical trials, as investigators try to further define its role in clinical practice. Results have been shared in many forums, including annual meetings of the American College of Rheumatology and European League Against Rheumatism, along with publications in the medical literature. These findings indicate that belimumab is effective in patients with more active disease who are able to wait up to 3 months for improvement. Additionally, it can be considered for patients with active disease despite therapy with NSAIDs, antimalarials, immunosuppressives, or corticosteroids, especially if the benefits of treatment outweigh its risks or if the patient is intolerant to steroids or immunosuppressives.

Additional file 1 contains an edited transcript of the full presentation.

## Competing interests

DJW has received consulting fees from Novo Nordisk, GSK, Pfizer, Lilly and UCB and been on advisory committees or review panels for UCB.

## Supplementary Material

Additional file 1 Click here for file

