# Supplementary material for: Clinical impact of biologic therapy in the treatment of SLE
Source: Arthritis Res Ther. 2013 Jul 11;15(Suppl 2):A5. doi: 10.1186/ar4223 (PMC3890904; doi:10.1186/ar4223)
Supplement: Additional file 1 [file ar4223-S1.pdf]

Treatment of SLE: Bridging the Gap from Clinical Trials to Practice

Presentation

Clinical impact of biologic therapy in the treatment of SLE

Daniel J Wallace

Clinical Professor of Medicine, Cedars-Sinai Medical Center, David Geffen School of Medicine at UCLA, Los Angeles, CA, USA

Abstract

Biologic therapies have generated substantial research interest for treating autoimmune diseases, resulting in several US Food and Drug Administration approvals. However, clinical trial results in systemic lupus erythematosus (SLE) populations have failed to meet efficacy expectations, primarily owing to methodological flaws in design, especially outcome measures. To address these issues, the US Food and Drug Administration published a guidance document for clinical trial design in SLE that included specific efficacy endpoints for measuring disease severity. Belimumab was the first biologic approved for SLE based on positive responses measured with the SLE Responder Index, which required a decrease in inflammation, no new organ domains being involved, and improvement in physician global assessment. Trials of other biologics have used other versions of SLE Responder Index responder indices. In addition to belimumab, a score of other biologic agents are being investigated, primarily those targeting T cells, B cells, complement, and tolerogen-targeting agents. Their development stages and clinical trial results vary. Belimumab has generated the greatest amount of clinical trials, as investigators try to further define its role in clinical practice. Results have been shared in many forums, including annual meetings of the American College of Rheumatology and European League Against Rheumatism, along with publications in the medical literature. These findings indicate that belimumab is effective in patients with more active disease who are able to wait up to 3 months for improvement. Additionally, it can be considered for patients with active disease despite therapy with NSAIDs, antimalarials, immunosuppressives, or corticosteroids, especially if the benefits of treatment outweigh its risks or if the patient is intolerant to steroids or immunosuppressives.

INTRODUCTION

The development of biologics for autoimmune disease has expanded the options for treating those diseases; however, investigations into their use for systemic lupus erythematosus (SLE) have not kept pace. Between 1982 and 2010, eight biologics failed to meet safety and efficacy endpoints in clinical trials in patients with SLE, including some biologics approved by the US Food and

Disclosures

About this presentation
This presentation was developed from an audio transcript of Dr. Wallace's presentation at the "Treatment of SLE: Bridging the Gap from Clinical Trials to Practice" symposium held during the Annual Congress of the American College of Rheumatology on November 11, 2012.

The transcript was formatted and edited by Cleveland Clinic and BioMed Central staff for clarity and conciseness, and was then reviewed, revised, and approved by Dr. Wallace.

Sponsorship
The Cleveland Clinic Foundation Center for Continuing Education acknowledges an educational grant for support of this activity from Human Genome Sciences.

Accreditation
The Cleveland Clinic Foundation Center for Continuing Education is accredited by the Accreditation Council for Continuing Medical Education to provide continuing medical education for physicians. The Cleveland Clinic Foundation Center for Continuing Education designates these (4) enduring activities for a maximum of **1.0** *AMA PRA Category 1 Credit*™. Physicians should claim only the credit commensurate with the extent of their participation in the activity. Participants claiming CME credit from this activity may submit the credit hours to the American Osteopathic Association for Category 2 credit.

To claim CME credit, visit www.ccfcme.org/SLECMEsupplement. CME credit may be obtained upon reading all four CME-certified presentations online.

Author disclosures
In accordance with the Standards for Commercial Support issued by the Accreditation Council for Continuing Medical Education (ACCME), The Cleveland Clinic Foundation Center for Continuing Education requires resolution of all faculty conflicts of interest to ensure CME activities are free of commercial bias. Dr. Wallace has indicated that he may have relationships which, in the context of his presentation, could be perceived as a potential conflict of interest. Dr. Wallace has received fees from UCB for consulting, teaching and speaking, and membership on advisory committee or review panels. He has also received fees from Astellas, Amgen, Abbott, Pfizer, Dynavax, TEVA, Cephalon, Lilly, Roche/Genentech, Sanofi, Janssen, Janus, Rigel, Human Genome Sciences, and Glaxo Smith Kline for consulting and membership on advisory board.

All other planners, CME staff, and content reviewers, have no relevant financial relationships to disclose.

This presentation has not been subject to peer review. The statements and opinions expressed herein are those of the author, who bears full responsibility for the content of this presentation.

Drug Administration (FDA) for other autoimmune diseases, including rituximab, abatacept, and anti-CD40L treatments. Methodologic flaws in the clinical trial design greatly contributed to the failed results. These primarily consisted of the following:

- Use of outcome measures not intended to be used in clinical trials with agents already shown to be effective in case series and retrospective studies.
- Underpowered studies relating to the numbers of patients enrolled.
- Primary outcome measures only available in English but were used in international trials, which confounded data.
- Investigators not adequately trained in certain measurements or measurements improperly vetted by the contract research/manufacturing organizations.
- Mandating corticosteroid regimens that are not used in clinical practice, creating an artificial environment.
- Multiple study groups received effective therapy, making it impossible to determine differences between groups.

To address these issues, the FDA published a guidance document for industry in June 2010 outlining steps for developing medical products in the treatment of SLE [1]. The requirements stipulated the following:

- Conducting two adequately controlled trials, preferably superiority trials with an open-label extension.
- Conducting a 1-year randomized controlled trial in patients who meet the American College of Rheumatology criteria for SLE [2].
- Stratification of patients by disease severity.
- Use of a valid disease activity index: British Isles Lupus Assessment Group (BILAG)-2004 is preferred; SLE Disease Activity Index (SLEDAI), European Consensus Lupu Activity Measure (ECLAM), and Systemic Lupus Activity Measure (SLAM) are acceptable.
- Use of defined terminology for major and partial clinical response, remission, reduction in flares, and time to flare.
- Corticosteroid use is best if minimized and steroid-sparing effects are carefully defined.
- Inclusion of patient-reported outcome measures.
- Selection of surrogate endpoints reasonably likely to predict clinical benefits based on epidemiologic, therapeutic, and pathophysiologic evidence.

A key element in the guidelines was the recommendation to select outcomes measures that integrate disease severity into treatment monitoring. In March 2011, belimumab was approved based on positive response rates from the BLISS-52 and BLISS-76 clinical trials using the SLE Responder Index (SRI), which included a decrease in inflammation, no new organ involvement, and improved physician global assessment. All three benchmarks had to be met to be considered a response [3,4].

The second furthest along agent for SLE is epratuzumab, which has shown efficacy in the phase 2 EMBLEM study [5]. Instead of using the SRI, investigators developed a responder classification called BICLA. This is an inverse of the SRI. Instead of reduced SLEDAI scores, it used ‘no worsening’ of the SLEDAI. Instead of no new BILAG A, it quantified the BILAG. The physician global assessment requirement was the same, but a stipulation was added that there could be no additional or increased dosing for immune suppressives, antimalarials, or steroids above baseline.

STUDIES OF BIOLOGIC AGENTS

Biologic agents being investigated for SLE are categorized based on their targets, whether T cells, B cells, complement, cytokines, the innate immune system, tolerogens, or cell surface receptor activation inhibition.

T cells

Abatacept is essentially the only approved rheumatism agent on the market that blocks T cells. Although abatacept did not meet its primary endpoint in either a lupus nephritis trial or a non-nephritis trial, certain subsets did respond to treatment, depending on the outcome measures used. The Immune Tolerance Network has a lupus nephritis trial nearing completion that is expected to show that abatacept is effective for at least certain subsets of patients with SLE [6]. It is highly likely that more trials with abatacept for SLE will be conducted to further investigate subsets that responded to therapy.

Several other T-cell inhibitors have been investigated:

- Two ICOS inhibitors are in clinical trials for SLE: ICOS-B7 RP1 and MEDI-570.
- Efalizumab, an anti-CD11a antibody immunosuppressant, was on the market for psoriasis but was withdrawn in 2012 because of concerns regarding risk of progressive multifocal leukoencephalopathy.
- CD40 ligand blockade has had two clinical trial failures, but several companies are working on reconfigurations of the CD40 ligand.

Abatacept is the furthest along in clinical trials among the T-cell inhibitors being investigated for SLE. Therapies focused on other T-cell pathways are many years from approval.

B cells

The B-cell-targeting therapies are divided into four categories based on their mechanism of action:

- Therapies that deplete or kill B cells (rituximab, ofatumumab, ocrelizumab).
- Therapies that block certain markers present in B-cell development including cytokines or proteins such as BLyS/BAFF/APRIL/TRAIL (belimumab, atacicept, tabalumab, blisibimod).
- Therapies that partially deplete and alter cell signaling (anti-CD22; for example, epratuzumab).
- Therapies that inhibit plasma cell activation (bortezomib, carfilzumib).

Mature B-cell-depleting agents (CD20 antagonists)

At present, three B-cell-depleting agents are being marketed; two have FDA indications for conditions other than SLE.

This presentation has not been subject to peer review. The statements and opinions expressed herein are those of the author, who bears full responsibility for the content of this presentation.

Rituximab rapidly kills B cells by antibody-dependent cell-mediated cytotoxicity, complement-dependent cytotoxicity, and apoptosis of B cells *in vitro*. Its efficacy is mediated through extensive B-cell depletion. Rituximab did not meet its primary outcome measures in poorly designed trials for both nephritis and non-nephritis SLE. Patients in both arms received effective high-dose steroid and immunosuppressive therapies, making it difficult to differentiate results. Nevertheless, results from case reports and retrospective analyses suggest that rituximab is effective for select cases of thrombocytopenia, thrombotic thrombocytopenic purpura, inflammatory arthritis, hemolytic anemia, and central nervous system vasculitis. An abstract presented at the 2012 American College of Rheumatology meeting showed that rituximab in combination with cyclophosphamide is an effective treatment for pediatric lupus.

Ofatumumab, a human anti-CD20 agent, is FDA approved for leukemia. Ofatumumab had positive results in a phase 3 rheumatoid arthritis trial and may have potential use in SLE: however, proposed trials are currently on hold.

Ocrelizumab development has been suspended because it did not meet the primary outcome measures in lupus nephritis and non-nephritis trials, owing to either increased infection rates or lack of efficacy.

B-cell-activating factors

Throughout the B-cell lifespan, receptors on B-cell membranes evolve and change, which means that treatments can be tailored to affect only certain types of B cells. Immature and naive B cells have some receptors not found in the bone marrow or in mature B cells. Among these are receptors for B-cell-activating factors that include BlyS/BAFF and APRIL. By blocking these activating factors, B-cell-activating factor drugs inhibit B-cell maturation.

In addition to belimumab, there are three important BlyS and/or APRIL-targeting agents: atacicept, tabalumab, and blisibimod.

Atacicept inhibits the binding of both BLyS and APRIL. In phase 1 studies of pharmacokinetic and biologic activity, atacicept has shown biologic effects on B cells when administered intravenously or subcutaneously [7]. Atacicept was well tolerated, with a favorable safety profile. However, a phase 3 trial was halted in October 2008 because of increased infection rates and neutropenia, especially in patients on mycophenolate [8]. Enrollment in a trial in 400 non-nephritis patients, which is really an immune suppressive withdrawal trial, has been completed. Results are expected to be presented in 2013.

Tabalumab is very similar to belimumab, with only two differences. Tabalumab blocks the activity of both soluble BLyS and membrane-bound BLyS, but is only available in a subcutaneous formulation as opposed to intravenous formulation. A phase 3 trial was started in 2011, and more than 1,000 patients have been enrolled, which is about 70% of the number needed.

Blisibimod has shown safety and some efficacy, specifically at higher doses in a subset of patients with very active disease [9]. It is now in a large phase 3 trial in which the entry criteria for patients include SLEDAI ≥10 who are also receiving prednisone.

Belimumab is an IgG_1 that blocks BLyS, which allows B-cell apoptosis to prevent differentiation of IgG plasma cells by preventing survival.

Partial B-cell-depleting agent (anti-CD22)

Epratuzumab is a CD22-targeting humanized mAb that targets the B-cell receptor and depletes B cells by 30 to 40% but seems to show efficacy without extensive B-cell reduction *in vitro*. Epratuzumab does not seem to cause complement-dependent cytotoxicity, although there is evidence of partial antibody-dependent cell-mediated cytotoxicity.

Results from ALLEVIATE trials (halted in 2006 due to supply issues) demonstrated steroid-sparing properties and improvements in quality of life [10]. In 5-year, open-label, follow-up studies of ALLEVIATE patients, they showed continued efficacy [11]. The EMBODY phase 3 trial has enrolled several hundred patients.

Data from the EMBLEM study suggest that this agent is most effective at meeting the BICLA response definition in the middle doses [5]. Both the ALLEVIATE and EMBODY trials have had favorable results, and publication is expected in 2013. This agent is the furthest along of any new molecular entities in development for SLE, and it appears to have an excellent safety profile.

Complement blockers

Research has identified the importance of the complement system in the pathogenesis of SLE. A mAb agent (eculizumab) that targets the C5 complement protein, thus blocking its generation of proinflammatory molecules, is FDA approved for paroxysmal hemoglobinuria. It has been investigated for SLE in a phase 1 single-dose study [12], and results suggest it may have a positive safety profile. There are case reports of the agent being effective in SLE, catastrophic antiphospholipid syndrome and antiphospholipid syndrome-associated recurrent pregnancy loss [13]. A study of patients with thrombotic thrombocytopenic purpura is in progress.

Cytokine-targeting strategies include inhibition of IL-6, IL-10, IL-12, IL-17, IL-18, IL-21, and IL-23. Three studies are investigating IL-6 inhibition, and an IL-21 inhibitor clinical trial is planned for next year. Anti-TNFs are also in the cytokine category, but they have not shown much promise for efficacy in SLE.

The IL-6 inhibitor tocilizumab is FDA approved for rheumatoid arthritis. A 2010 study in a small number of patients with SLE (n = 16) showed that this agent produced improvements in clinical and serologic responses, including decreases in acute-phase reactants

This presentation has not been subject to peer review. The statements and opinions expressed herein are those of the author, who bears full responsibility for the content of this presentation.

and anti-dsDNA antibodies, improvement in overall disease activity, and normalization of circulating B cells [14]. However, the incidence of neutropenia and infections prompted some concern. Overall, the study was too small to draw clinical conclusions. Nevertheless, the results do indicate that IL-6 plays an important role in lupus pathogenesis, making it an attractive target for therapeutic intervention. At present, several anti-IL-6 SLE trials are underway.

Tolerogen-targeting agents

These antigens are another target, and several immunomodulator drugs targeting them are being investigated. Two have shown promise – laquinimod and rigerimod. In phase 2 studies of more than 3,000 patients with Crohn's disease and multiple sclerosis, laquinimod has had favorable results [15,16]. Rigermod and lupuzor/fogerimod are two preparations of the same chemical, and both have demonstrated safety and modest benefits in phase 1 and 2 studies. A phase 3 trial is planned. Laquinimod works by shifting immune modulation towards Th2; fogerimod promotes tolerance by promoting autophagy by preventing the proliferation of $CD4^+$ T cells, and promoting secretion of IL-10 and decreasing anti-DNA in SLE [17].

Interferons

Research on interferons has identified several important roles that they play in the pathogenesis of SLE. Several connections have been identified between interferon and SLE:

- Serum IFNα levels are elevated in patients with SLE [18].
- Increased expression of type I interferon-induced genes in blood and involved tissues in SLE [19].
- Correlation between interferon levels and expression of type I interferon-induced genes and SLE activity [18,20].
- Development of SLE in patients undergoing IFNα treatment [21].

The implication is that inhibition of IFNα may provide therapeutic benefit to patients with SLE, leading to an interest in agents that block interferon and toll-like receptors. Results from research targeting IFNa[ED] or IFNγ have thus far been disappointing. Several agents targeting other cell-signaling pathways in the immune system are also in development, but it is too early to draw any conclusions.

Belimumab

Belimumab (Benlysta) is the only biologic FDA approved to treat SLE. It was approved on 8 March 2011 for the reduction of disease activity in adult patients with active, autoantibody-positive SLE who are receiving standard therapy. Approval was based on two pivotal phase 3 trials, along with safety data from these trials and a phase 2 trial with an open-label extension [4,22,23].

Recent study data

Belimumab has not been evaluated in patients with lupus nephritis or severe central nervous system active disease or in combination with other biologics or intravenous cyclophosphamide. Since 2010, an estimated 10,000 people have received belimumab, and no significant adverse event profile has emerged. There has been one mortality from anaphylactic reactions in a woman, but she had multiple comorbidities and no change in drug labeling was necessitated.

Table 1 presents recently published data on belimumab in patients with SLE. Those results have led to several clinical insights.

The 2011 American College of Rheumatology meeting presented the following:

- Renal outcomes in a small subgroup of patients with acute lupus nephritis suggest that they may have outcomes similar to those with active SLE but without nephritis, which offers support for an initiation of a randomized trial in patients with nephritis.
- Adverse events have not been statistically different from placebo, including effects on pregnancies.
- Improvements in both fatigue and quality of life have been observed, and the results correlate well with BILAG domains.
- Patients who achieve SRI response have fewer BILAG organ domain involvement, fewer flares, and significant improvements in C3 and anti-dsDNA.

The 2012 European League Against Rheumatology meeting presented the following:

- The superiority of combined mycophenolate and belimumab versus monotherapy with either.
- Meaningful early clinical improvement occurs in the first 3 months.
- Decreased flares in both SLEDAI and BILAG.

Peer-reviewed medical literature presents the following:

- Sicker patients tend to do better.
- Musculoskeletal and mucocutaneous domains improve the most, although they had the most subjects in those domains.
- Patients tolerate inactivated vaccinations (but not live-virus vaccines) very well, although they may be slightly less effective than in healthy controls.
- Impressive improvements were noted for several immunologic endpoints.
- Long-term steroid sparing and immune suppression continues for at least 6 years in patients during an open-label, follow-up study.

The 2012 American College of Rheumatology meeting presented the following:

- Phase 2 trials showed encouraging results in patients with Sjogren's syndrome, offering some justification for more controlled trials of this off-label indication.
- Nonindustry-sponsored research showed that the drug works as early as 3 months and is effective in African Americans, a population that is currently being studied separately.

This presentation has not been subject to peer review. The statements and opinions expressed herein are those of the author, who bears full responsibility for the content of this presentation.

Table 1. Belimumab trials for systemic lupus erythematosus: *post hoc* data analyses

Author/citation	Details	Patient population	Results/comments
**American College of Rheumatology 2011**			
Dooley and colleagues [24]	Three phase 3	333	Patients with acute lupus nephritis had results similar to those without nephritis
Wallace and colleagues [25]	Phase 2/3	3,000	Rates of serious infection, malignancy, mortality, and infusion reactions similar to placebo; 70% of pregnancies successful, rate same in placebo recipients
Strand and colleagues [26]	BLISS-52, BLISS-76	1,684	Improvements in fatigue and QOL, correlate with BILAG domains
Furie and colleagues [27]	BLISS-52, BLISS-76	1,684	SRI responders (n = 761) had significantly improved BILAG organ domains, flare count, changes in C3, anti-dsDNA
**European League Against Rheumatology 2012**			
Schneider and colleagues [28]	BLISS-52, BLISS-76	189	Combination of MMF + belimumab superior to belimumab without MMF
Houssiau and colleagues [29]	BLISS-52, BLISS-76	189	In patients with nephritis, renal flare rates were less with combined belimumab + MMF
Doria and colleagues [30]	BLISS-52, BLISS-76		Meaningful improvement occurs in first 3 months in serology, self-reports, and inflammation metrics
Petri and colleagues [31]	Phase 2 LBSL02/99 Study Group	449	SLE Flare Index and BILAG A–B flares significantly lower in belimumab-treated patients
**Journal articles**			
van Vollenhoven and colleagues [32]	BLISS-52, BLISS-76	1,684	Greater therapeutic effect vs. standard treatment in patients with higher disease activity, anti-dsDNA positivity, low complement, steroid treatment at baseline
Manzi and colleagues [33]	BLISS-52, BLISS-76	1,684	Greatest improvements vs. placebo in musculoskeletal, mucocutaneous domains (all statistically significant)
Chatham and colleagues [34]	BLISS-76	204	Vaccinations: pneumococcal, influenza, tetanus titers satisfactory in belimumab group, slightly lower than controls
Stohl and colleagues [35]	BLISS-52, BLISS-76	1,684	Significantly decreased IgG, autoantibodies, naive and activated B cells, plasma cells; improved C3/C4
Merrill and colleagues [26]	Phase 2 LBSL02/99 Study Group	449	6-year open-label follow-up showed continued safety and efficacy with steroid/immune-suppressive sparing properties
**American College of Rheumatology 2012 abstracts**			
Mariette and colleagues [37], De Vital and colleagues [38]	Phase 2, Beliss study	60	Sjogren's syndrome: improved glandular swelling, ESSDAI scores
Buyon and colleagues [39], Kim and colleagues [40]		106	Nonindustry sponsored; improved diseases activity noted at 3 months
Collins and colleagues [41]	Cohort study	58	African Americans with SLE: most had clinical improvements at 6 months
van Vollenhoven and colleagues [42], Petri and colleagues [43], Clarke and colleagues [44]	BLISS-52, BLISS-76	562 (Placebo group)	Flares in those with SLEDAI >12, organ involvement, low C3, high anti-dsDNA, high C-reactive protein, proteinuria among placebo recipients
Cail and colleagues [45]		118	Bioavailability, pharmacokinetics: results similar for subcutaneous vs. intravenous
Merrill and colleagues [46]	Open-label extension of phase 2 RCT	190	Sustained safety, efficacy seen at 7 years

BILAG, British Isles Lupus Assessment Group; ESSDAI, EULAR Sjögren's Syndrome Disease Activity Index; MMF, mycophenolate mofetil; QOL, quality of life; RCT, randomized controlled trial; SLE, systemic lupus erythematosus; SLEDAI, SLE Disease Activity Index; SRI, SLE Responder Index.

This presentation has not been subject to peer review. The statements and opinions expressed herein are those of the author, who bears full responsibility for the content of this presentation.

- Subcutaneous administration is just as effective as intravenous.
- Long-term safety and efficacy is sustained over a 7-year period.

In addition, several phase 4 studies in patients with SLE have been registered with the FDA. These include trials of the subcutaneous versus intravenous formulations, African Americans, lupus nephritis, and pediatric lupus. Other efforts include creating a pregnancy database and emphasizing the mental health inventories such as suicidality, depression, and anxiety. Trials with non-SLE patients that are in development include idiopathic thrombocytopenic purpura, Sjogren's syndrome, vasculitis, glomerulonephritis, and myasthenia gravis.

Clinical implications

Belimumab is an effective drug in patients with active SLE (those who disease is most active respond best) who can wait up to 3 months for improvement. Belimumab can be considered for patients with active disease despite therapy with NSAIDs, antimalarials, immunosuppressives, or corticosteroids, especially if the potential benefits of treatment with belimumab outweigh the known risks of long-term treatment with corticosteroids (for example, avascular necrosis, diabetes) or if the patient is intolerant to immunosuppressives (for example, experiences gastrointestinal or hematologic issues with azathioprine or mycophenolate mofetil).

ABBREVIATIONS

BILAG, British Isles Lupus Assessment Group; FDA, US Food and Drug Administration; IFN, interferon; IL, interleukin; mAb, monoclonal antibody; NSAID, nonsteroidal antiinflammatory drug; SLE, systemic lupus erythematosus; SLEDAI, SLE Disease Activity Index; SRI, SLE Responder Index; TNF, tumor necrosis factor.

REFERENCES

1. **Guidance for Industry: Systemic Lupus Erythematosus – Developing Medical Products for Treatment** [http://www.fda.gov/downloads/Drugs/GuidanceComplianceRegulatoryInformation/Guidances/ucm072063.pdf]
2. Hahn BH, McMahon MA, Wilkinson A, Wallace WD, Daikh DI, Fitzgerald JD, Karpouzas GA, Merrill JT, Wallace DJ, Yazdany J, Ramsey-Goldman R, Singh K, Khalighi M, Choi S, Gogia M, Kafaja S, Kamgar M, Lau C, Martin WJ, Parikh S, Peng J, Rastogi A, Chen W, Grossman JM: **American College of Rheumatology guidelines for screening, treatment, and management of lupus nephritis.** *Arthritis Care Res* 2012, **64:**797-808.
3. Furie R, Nicholls K, Cheng T-T, Houssiau F, Burgos-Vargas R, Chen S-L, Aranda R, Meadows-Shropshire S, Kinaszczuk M, Merrill JT: **Efficacy and safety of abatacept over 12 months in patients with lupus nephritis: results from a multicenter, randomized, double-blind, placebo-controlled phase II/III study [abstract 2469].** In *ACR/ARHP Scientific Meeting; November 5-9, 2011; Chicago, IL.*
4. Navarra SV, Guzman RM, Gallacher AE, Hall S, Levy RA, Jimenez RE, Li EK, Thomas M, Kim HY, Leon MG, Tanasescu C, Nasonov E, Lan JL, Pineda L, Zhong ZJ, Freimuth W, Petri MA; BLISS-52 Study Group: **Efficacy and safety of belimumab in patients with active systemic lupus erythematosus: a randomised, placebo-controlled, phase 3 trial.** *Lancet* 2011, **377:**721-731.
5. Wallace DJ, Kalunian K, Petri MA, Strand V, Houssiau FA, Pike M, Kalgallen B, Bongardt S, Barry A, Kelley L, Gordon L: **Efficacy and safety of epratuzumab in patients with moderate/severe active systemic lupus erythematosus: results from EMBLEM, a phase IIb, randomized, double-blind, placebo-controlled multicentre study.** *Ann Rheum Dis* 2013. [Epub ahead of print]
6. Wofsy D, Hillson JL, Diamond B: **Abatacept for lupus nephritis: alternative definitions of complete response support conflicting conclusions.** *Arthritis Rheum* 2012, **64:**3660-3665.
7. Dall'Era M, Chakravarty E, Genovese M, Wallace D, Kavanaugh A, Kalunian K, Dhar P, Pena-Rossi C, Wofsy D: **Trial of atacicept in patients with systemic lupus erythematosus**. *Arthritis Rheum* 2006, **54:**4042-4043.
8. Ginzler EM, Wax S, Rajeswaran A, Copt S, Hillson J, Ramos E, Singer NG: **Atacicept in combination with MMF and corticosteroids in lupus nephritis: results of a prematurely terminated trial.** *Arthritis Res Ther* 2012, **14:**R33.
9. Furie RA, et al. **Blisibimod, an inhibitor of B cell activating factor in patients with moderate to severe systemic lupus erythematosus [late breaking abstract L6].** *Arthritis Rheum* 2012, **64**:4169.
10. Wallace D, Gordon C, Strand V, Hobbs K, Petri M, Kalunian K, Houssiau F, Tak PP, Isenberg DA, Kelley L, Kilgallen B, Barry AN, Wegener WA, Goldenberg DM: **Efficacy and safety of epratuzumab in patients with moderate/severe flaring systemic lupus erythematosus: results from two ransomized, double-blind, placebo-controlled, multicentre studies (ALLEVIATE) and follo-up.** *Rheumatology (Oxford)* 2013 [Epub ahead of print].
11. Hobbs K, Wallace DJ, Strand V, Kalunian K, Kilgallen B, Bongardt S, Wegener WA, Goldenberg DM: **Safety and efficacy of epratuzumab in open-label extension study (SL006).** *Arthritis Rheum* 2012, **64(Suppl):**S276-S277.
12. Furie R, Matis L, Rollins S*:* **A single dose, placebo-controlled, double blind, phase I study of the humanized anti-C5 antibody hbG1.1 in patients with systemic lupus erythematosus.** In *ACR/ARHP Scientific Meeting, October 16-21 2009; Philadelphia, PA*.
13. Velik-Salchner C, Lederer W, Wiedermann F: **Eculizumab and renal transplantation in a patient with catastrophic antiphospholipid syndrome: effect of heparin on complement activation.** *Lupus* 2011, **20:**772.
14. Illei GG, Shirota Y, Yarboro CH, Daruwalla J, Tackey E, Takada K, Fleisher T, Balow JE, Lipsky PE: **Tocilizumab in systemic lupus erythematosus: data on safety, preliminary efficacy, and impact on circulating plasma cells from an open-label phase I dosage-escalation study.** *Arthritis Rheum* 2010, **62:**542-552.
15. Comi G, Jeffery D, Kappos L, Montalban X, Boyko A, Rocca MA, Filippi M; ALLEGRO Study Group: **Placebo-controlled trial of oral laquinimod for multiple sclerosis.** *N Engl J Med* 2012, **366:**1000-1009.
16. Comi G, Pulizzi A, Rovaris M, Abramsky O, Arbizu T, Boiko A, Gold R, Havrdova E, Komoly S, Selmaj K, Sharrack B, Filippi M; LAQ/5062 Study Group: **Effect of laquinimod on MRI-monitored disease activity in patients with relapsing-remitting multiple sclerosis: a multicentre, randomised, double-blind, placebo-controlled phase IIb study.** *Lancet* 2008, **371:**2085-2092.
17. Zimmer R, Scherbarth HR, Rillo OL, Gomez-Reino JJ, Muller S: **Lupuzor/P140 peptide in patients with systemic lupus erythematosus: a randomised, double-blind, placebo-controlled phase IIb clinical trial.** *Ann Rheum Dis* 2012. [Epub ahead of print]
18. Hooks JJ, Moutsopoulos HM, Geis SA, Stahl NI, Decker JL, Notkins AL: **Immune interferon in the circulation of patients with autoimmune disease.** *N Engl J Med* 1979, **301:**5-8.
19. Crow MK: **Type I interferon and autoimmune disease.** *Autoimmunity* 2003, **36:**445-446.
20. Dall'era MC, Cardarelli PM, Preston BT, Witte A, Davis JC Jr: **Type I interferon correlates with serological and clinical manifestations of SLE.** *Ann Rheum Dis* 2005, **64:**1692-1697.
21. Ioannou Y, Isenberg DA: **Current evidence for the induction of autoimmune rheumatic manifestations by cytokine therapy.** *Arthritis Rheum* 2000, **43:**1431-1442.
22. Jacobi AM**,** Huang W**,** Wang T, Freimuth W**,** Sanz I**,** Furie R**,** Mackay M**,** Aranow C, Diamond B, Davidson A: **Effect of long-term belimumab treatment on B cells in systemic lupus erythematosus: extension of a phase II, double-blind, placebo-controlled, dose-ranging study.** *Arthritis Rheum* 2010, **62:**201-210.

23. Wallace DJ, Stohl W, Furie RA, Lisse JR, McKay JD, Merrill JT, Petri MA, Ginzler EM, Chatham WW, McCune WJ, Fernandez V, Chevrier MR, Zhong ZJ, Freimuth WW: **A phase II, randomized, double-blind, placebo-controlled, dose-ranging study of belimumab in patients with active systemic lupus erythematosus.** *Arthritis Rheum* 2009, **61:**1168-1178.
24. Dooley MA, Houssiau F, Aranow C, D'Cruz DP, Askanase A, Roth DA, Zhong ZJ, Cooper S, Freimuth WW, Ginzler EM; BLISS-52 and BLISS-76 Study Groups: **Effect of belimumab treatment on renal outcomes: results from the phase 3 belimumab clinical trials in patients with SLE.** *Lupus* 2013, **22:**63-72.
25. Wallace D, Navarra S, Petri M, Gallacher A, Thomas M, Furie R, Levy R, van Vollenhoven R, Cooper S, Zhong Z, Freimuth W, Cervera R; BLISS-52 and BLISS-76 and LBSL02 Study Groups: **Safety profile of belimumab: pooled data from placebo-controlled phase 2 and 3 studies in patients with systemic lupus erythematosus.** *Lupus* 2013, **22:**144-154.
26. Strand V, Cooper S, Zhong ZJ, Dennis G: **Responders in the phase 3 belimumab clinical trials in patients with systemic lupus erythematosus reported improvements in fatigue and health-related quality of life at week 52 [abstract 1369].** In *ACR/ARHP Scientific Meeting; November 5-9 2011; Chicago, IL*.
27. Furie RA, Zhong ZJ, Freimuth W, Petri M: **Clinical and laboratory correlates in responders (by the Systemic Lupus Erythematosus Responder Index) in phase 3 belimumab clinical trials [abstract 1367].** In *ACR/ARHP Scientific Meeting; November 5-9 2011; Chicago, IL.*
28. Schneider M, Buyon J, Dooley MA, Ginzler EM, Cooper S, Zhong JZ, Keenan GF, Merrill JT: **Impact of mycophenolate mofetil and/or corticosteroid treatment on outcomes of belimumab treatment in SLE.** *Ann Rheum Dis* 2012, **71(Suppl 3):**537.
29. Houssiau F, Dooley MA, Aranow C, D'Cruz D, Askanase A, Roth DA, Cooper S, Zhong JZ, Freimuth W, Ginzler EM; BLISS-52/-76 Study Groups: **Post-hoc analysis to assess effect of belimumab in patients on mycophenolate mofetil with renal manifestations at baseline.** *Ann Rheum Dis* 2012, **71(Suppl 3):**536.
30. Doria A, Sanchez-Guerrero J, Tegzová D, Ginzler EM, Zhong JZ, Dennis G, Wallace DJ: **Early clinical improvement in SLE patients treated with belimumab.** *Ann Rheum Dis* 2012, **71(Suppl 3):**535.
31. Petri M, Furie R, Merrill J, Wallace D, Stohl W, Chatham W, Weinstein A, McKay J, Ginzler E, Zhong Z, Pineda L, Klein J, Freimuth W; LBSL02/99 Study Group**. Six-year experience with belimumab in patients with SLE.** *Ann Rheum Dis* 2011, **70(Suppl 3):**314.
32. van Vollenhoven RF, Petri MA, Cervera R, Roth DA, Ji BN, Kleoudis CS, Zhong ZJ, Freimuth W: **Belimumab in the treatment of systemic lupus erythematosus: high disease activity predictors of response.** *Ann Rheum Dis* 2012, **71:**1343-1349.
33. Manzi S, Sánchez-Guerrero J, Merrill JT, Furie R, Gladman D, Navarra SV, Ginzler EM, D'Cruz DP, Doria A, Cooper S, Zhong ZJ, Hough D, Freimuth W, Petri MA; BLISS-52 and BLISS-76 Study Groups: **Effects of belimumab, a B lymphocyte stimulator-specific inhibitor, on disease activity across multiple organ domains in patients with systemic lupus erythematosus: combined results from two phase III trials.** *Ann Rheum Dis* 2012, **71:**1833-1838.
34. Chatham WW, Wallace DJ, Stohl W, Latinis KM, Manzi S, McCune WJ, Tegzova D, McKay JD, Avila-Armengol HE, Utset TO, Zhong ZJ, Hough DR, Freimuth WW, Migone TS; BLISS-76 Study Group: **Effect of belimumab on vaccine antigen antibodies to influenza, pneumococcal, and tetanus vaccines in patients with systemic lupus erythematosus in the BLISS-76 trial.** *J Rheumatol* 2012, **39:**1632-1640.
35. Stohl W, Hiepe F, Latinis KM, Thomas M, Scheinberg MA, Clarke A, Aranow C, Wellborne FR, Abud-Mendoza C, Hough DR, Pineda L, Migone TS, Zhong ZJ, Freimuth WW, Chatham WW; BLISS-52 Study Group; BLISS-76 Study Group: **Belimumab reduces autoantibodies, normalizes low complement levels, and reduces select B cell populations in patients with systemic lupus erythematosus.** *Arthritis Rheum* 2012, **64:**2328-2337.
36. Merrill JT, Ginzler EM, Wallace DJ, McKay JD, Lisse JR, Aranow C, Wellborne FR, Burnette M, Condemi J, Zhong ZJ, Pineda L, Klein J, Freimuth WW; LBSL02/99 Study Group: **Long-term safety profile of belimumab plus standard therapy in patients with systemic lupus erythematosus.** *Arthritis Rheum* 2012, **64:**3364-3373.
37. Mariette X, Quartuccio L, Seror RL, Salvin S, Desmoulins F, Fabris M, Villeneuve S, Ravaud P, De Vita S: **Results of the Beliss Study, the first open phase 2 study of belimumab in primary Sjogren's syndrome [abstract 2555].** In *ACR/ARHP Scientific Meeting; November 9-14 2012; Washington, DC*.
38. De Vital S, le Seror R, Quartuccio L, Desmoulins F, Salvin S, Baron G, Fabris M, Ravaud P, Isola M, Mariette X: **Efficacy of belimumab on non-malignant parotid swelling and systemic manifestations of Sjogren's Syndrome: results of the Beliss study [abstract 2189].** In *ACR/ARHP Scientific Meeting; November 9-14 2012; Washington, DC*.
39. Buyon P, Belmont HM, Franks AG, Furie R, Kamen DL, Manzi S, Petri M, Ramsey-Goldman R, Tseng CE, van Vollenhoven RF, Wallace D, Askanas A: **Favorable response to belimumab at three months [abstract 1417].** In *ACR/ARHP Scientific Meeting; November 9-14 2012; Washington, DC*.
40. Kim SS, Pavri T, Kirou KA, Salmon J, Erkan D: **Real world experience with belimumab in the management of systemic lupus erythematosus (SLE): a single center, observational, post-marketing study [abstract 2246].** In *ACR/ARHP Scientific Meeting; November 9-14 2012; Washington, DC*.
41. Collins CE, Narayanan S, Dall'Era M, Dennis G, Oglesby A, McGuire MB, Pappu R, Molta CT, Keenan G: **Outcomes associated with belimumab in Black/African American patients with systemic lupus erythematous in clinical practice settings in the United States [abstract 2617].** In *ACR/ARHP Scientific Meeting; November 9-14, 2012; Washington, DC*.
42. van Vollenhoven RF, Petri MA, Levy RA, Navarra SV, Buyon JP, Zhong ZJ, Freimuth WW, Cervera R: **Predictors of systemic lupus** erythematosus **flares: baseline disease activity and demographic characteristics from the combined placebo groups in the phase 3 belimumab trials [abstract 614].** In *ACR/ARHP Scientific Meeting; November 9-14 2012; Washington, DC*.
43. Petri MA, van Vollenhoven RF, Levy RA, Navarra SV, Cervera R, Zhong ZF, Freimuth WW, Buyon JP: **Baseline laboratory characteristics from the combined placebo groups in the phase 3 belimumab trials are predictive of severe flare at 52 weeks [abstract 615].** In *ACR/ARHP Scientific Meeting; November 9-14 2012; Washington, DC*.
44. Clarke AE, Manzi S, Petri MA, Furie R, van Vollenhoven RF, Cooper S, Zhong ZJ, Freimuth WW, Weinstein A: **Efficacy of belimumab in systemic lupus erythematosus patients with high baseline disease activity [abstract 2241].** In *ACR/ARHP Scientific Meeting; November 9-14 2012; Washington, DC*.
45. Cai1 W, Chen C, Zhong ZJ, Freimuth WW, Lewis W, Subich D: **Bioavailability, pharmacokinetics, and safety of belimumab administered subcutaneously in healthy subjects [abstract 616].** In *ACR/ARHP Scientific Meeting; November 9-14 2012; Washington, DC*.
46. Merrill JT, Furie RA, Wallace DJ, Stohl W, Chatham WW, Weinstein A, McKay JD, Ginzler EM, Zhong AJ, Freimuth WW, Petri MA: **Sustained disease improvement and safety profile over 1745 patient-year experience (7 years) with belimumab in systemic lupus erythematosus patients [abstract 2621].** In *ACR/ARHP Scientific Meeting; November 9-14 2012; Washington, DC.*

This presentation has not been subject to peer review. The statements and opinions expressed herein are those of the author, who bears full responsibility for the content of this presentation.
